# A Combined Approach for the Aesthetic Management of Stained Enamel Opacities: External Bleaching Followed by Resin Infiltration

**DOI:** 10.1155/2018/1605842

**Published:** 2018-07-09

**Authors:** O. Marouane, N. Douki, F. Chtioui

**Affiliations:** Restorative Dentistry, Dental Surgery Department, Sahloul University Hospital, Sousse, Tunisia

## Abstract

Stained enamel opacities are frequently encountered in dental practice. However, due to the risk of unaesthetic outcome, managing such lesions by resin infiltration techniques alone is not advised. Therefore, performing external bleaching before resin infiltration procedure is mandatory to eliminate stains from the hypomineralized lesions in order to aesthetically infiltrate them. In this work, we describe clinical cases in which external bleaching and resin infiltration techniques were used for managing stained enamel hypomineralized lesions related to traumatic dental injuries and molar incisor hypomineralization. Despite the fact that this approach has some limitations, it could be concluded that external bleaching associated with the resin infiltration technique shows promising results to aesthetically manage stained enamel opacities when the stain is totally removed after bleaching.

## 1. Introduction

Stained enamel opacities (SEO) are frequently observed in our dental practice. They can be defined as aberrations of the quality of dental enamel which ranges clinically from yellow to brown due to its pigmentation while it appears to be histologically hypomineralized. SEO differs from unstained enamel opacities as they show a bright opaqueness in the absence of pigments in the hypomineralized enamel [1, 2].

The underlying aetiologies are multiple, but the categories are mainly twofold: posteruptive and preeruptive damages. A preeruptive damage is a consequence of a dysfunction in the enamel organ due to a variety of agents and leading to various pathological conditions such as fluorosis, traumatic hypomineralisation, and molar-incisor hypomineralization (MIH) [3–5]. Posteruptive damage of the enamel is, however, a result of the early manifestation of the carious process leading to lesion called brown spot [6, 7].

From an aesthetic point of view, treating SEO conservatively using the resin infiltration procedure is very complex. Indeed, it has been shown that performing resin infiltration on SEO is not effective. It induces a stain reemergence with an unpleasant aesthetic outcome [2]. For this reason, aesthetic management of SEO using resin infiltration technique alone is avoided and more invasive treatment options such as composite restorations, veneers, or crowns are used to correct the aesthetic defect [7–9]. Biologically, resin infiltration technique is a therapeutic of choice to aesthetically manage SEO. Indeed, resin infiltration allows to correct the aesthetic defect in a microinvasive way with improving the mechanical properties of such lesions [5, 7, 10, 11].

To date, there are only few papers reporting the aesthetic management of SEO using the resin infiltration technique.

In the aim of improving the aesthetic appearance of three patients, we describe in this paper the aesthetic management of cases presenting SEO using external bleaching followed by resin infiltration.

## 2. Case Report

Three patients between 12 and 23 years of age, with SEO located in their maxillary incisors, were referred to the Dental Medicine Department at Sahloul University Hospital, Sousse, Tunisia. All patients reported the discomfort caused by the presence of opacities on their anterior teeth as they affected their self-esteem and social lives.

**Figure 1:**
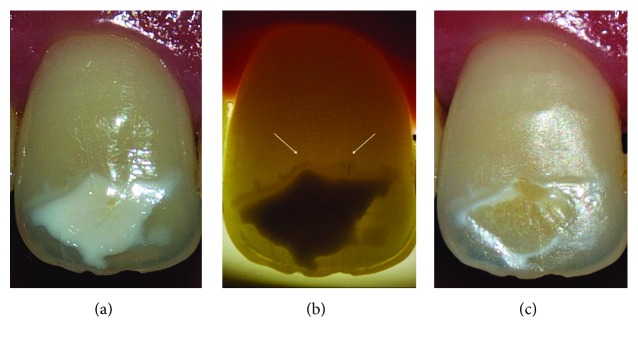
Stained enamel opacity related to traumatic dental injuries (a). The underestimation of the presence of stain into the lesion have led to unaesthetic outcomes after performing resin infiltration (c). Note the presence of enamel cracks (white arrow), observed under transillumination, which could constitute a possible pathway for chromatogenic substances (b). This case is considered a failure.

**Figure 2:**
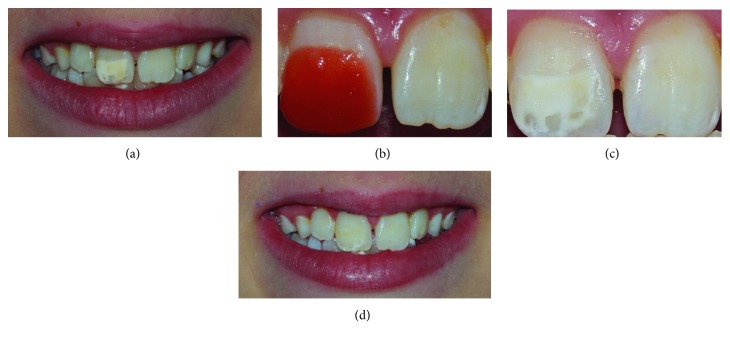
Yellowish lesion related to molar incisor hypomineralization (a). The combination of in-office bleaching (38% H_2_O_2_) (b) applied on a single visit for 15 min (c) and resin infiltration procedure were sufficient to end up with an esthetically satisfying result. By the end of the infiltration step, the incisal part of the lesion was not completely infiltrated probably due to excessive depth of the lesion at this area (d).

**Figure 3:**
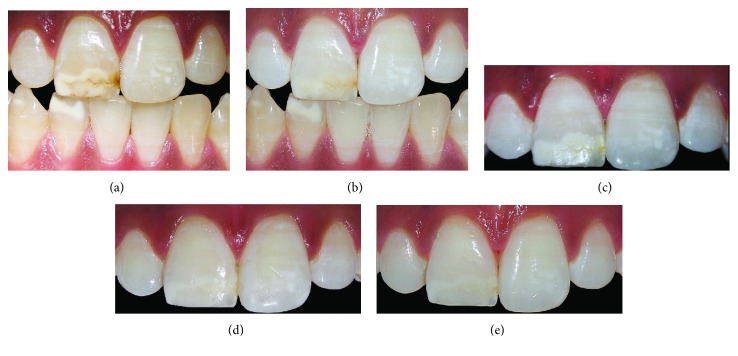
Brownish lesion related to molar incisor hypomineralization (a). After one week of at-home whitening using 10% carbamide peroxide gel (b), the stain was partially removed and has disappeared completely by the end of the bleaching treatment (c). Immediate result after performing resin infiltration (d). 3 months after treatment (e). Significant improvement in the aesthetic appearance of this patient's teeth was achieved, and the case was thus considered to be a success.

Meticulous clinical examination helped us to properly set the aetiology for each lesion. In the first case, SEO related to traumatic dental injury (TDI) (Figure 1(a)), and in the remaining cases, SEO related to molar incisor hypomineralization (MIH) (Figures 2(a) and 3(a)).

In order to aesthetically manage these SEO, the treatment consisted on external bleaching followed by resin infiltration. The idea behind this approach is to, first of all, remove the stain then perform resin infiltration. However, for the first patient (Figure 1(a)), due to the lack of evidence of staining, solely, resin infiltration procedure was adopted.

After obtaining a full written consent, each patient received an external bleaching procedure which was performed as follows:

- The second patient applied in-office bleaching gel (38% H_2_O_2_ Opalescence Xtra Boost; Ultradent) according to the manufacturer's instructions for 15 min selectively on the stained enamel opacity on a single session (Figure 2(b)).
- The third patient was instructed to use a whitening gel which contained 10% carbamide peroxide 10% (Philips Zoom NiteWhite, Discus Dental, Stamford, USA) (Figures 3(b) and 3(c)) delivered via a custom-fitting mouth tray for 21 nights.

Two weeks after completing the whitening treatment, resin infiltration procedure was performed. The surface layer was etched at first by applying of a 15% hydrochloric acid gel (Icon-etch) for 120 seconds followed by a water rinse for 30 seconds.

Then, the lesion was dried out with ethanol solution for 30 seconds (Icon-dry). After this step, the resin (Icon-infiltrant) was applied gently in a circular motion for 3 minutes then light-cured for 40 seconds. Finally, the excess was removed and the enamel surface was polished.

Except for the first patient, where the infiltration technique produced unsatisfactory and unaesthetic results, a considerable esthetic improvement was achieved in the other cases.

## 3. Discussion

As presented in these cases, enamel opacities may be discoloured with an appearance ranging from stain hardly distinguishable (Figure 1(a)) to yellow (Figure 2(a)) or brown (Figure 3(a)).

The histological structure of MIH or enamel opacity related to TDI has been described in the literature in a number of papers. On a microscopic scale, these lesions exhibit disorganised enamel prisms, separated with gaps containing a protein-rich matrix [1, 12–14]. Moreover, these lesions present a lower hardness and higher porosity than sound enamel [15, 16]. This histological structure implicates their weak mechanical properties and explains why these lesions often crack (Figure 1(b)) [14–16].

As presented, in the first case (Figures 1(a)–1(c)), performing resin infiltration on SEO must be avoided. In fact, the infiltration itself is probably not altered, but since the colour masking effect is related to the refractive index of the low viscosity resin, it has no effect on the brownish staining that still remained at the end of the treatment. Therefore, even if the stain is hardly distinguishable or questionable, it may reemerge following the infiltration procedure leading to an unaesthetic outcome [2].

The latter however once observed at the first patient cannot be attributed solely to the discolouring agents trapped within the enamel. In fact, the camphorquinone (CQ) used as a photoinitiator in the resin presents a yellowish colour. Yet during light curing, the CQ loses this colour as it infiltrates the lesion. In case of incomplete consumption of CQ, the resin may remain yellowish which may indicate the presence of unconsumed CQ [17, 18].

As illustrated in the second and third cases, removing stains from enamel opacities using external bleaching provides an essential pretreatment step to aesthetically manage SEO before proceeding with the resin infiltration. The aim of the bleaching procedure is to remove the stains within and acquire the desired esthetic outcome. Despite the fact that no differences in treatment efficacy were detected between at-home bleaching and in-office bleaching, other considerations must be, yet, taken before choosing one or the other [19].

In the second case, we performed a focal in-office bleaching for several reasons: firstly, due to the young age of the patient and, secondly, due to the mild colouration (yellow) of the opacity in association with correct brightness of the remaining teeth (Figures 2(a) and 2(b)) [20].

Otherwise, as was performed on the third patient, at-home whitening treatment containing 10% carbamide peroxide may also be effective to completely remove stains from the enamel opacity.

In addition to masking enamel opacities, resin infiltration technique also produces a positive side effect. It genuinely leads to an increase in the enamel surface hardness reinforcing its weakened histological structure that was additionally affected by the external bleaching [21].

After performing external bleaching, the adhesion of resin to the enamel becomes compromised for up to 14 days, and so a two- to three-week waiting period is necessary. Moreover, it was recently demonstrated that performing resin infiltration, directly after a bleaching procedure, affects negatively the penetration depth of the infiltrant [21].

## 4. Conclusion

To aesthetically manage stained enamel opacities, the stain must be totally removed by performing an external bleaching. Once the latter is successfully achieved, resin infiltration technique may subsequently allow a significant improvement in the appearance of teeth in a relatively short working time.

Although the results in the cases described in this work showed a partial disappearance of the stained lesions, the treatment outcome may be, all in all, considered successful.

However, the suggested treatment protocol might present some limitations. A correct inspection of these lesions remains essential to make a proper diagnosis and to propose a correct treatment plan. Although the final results flowing from the present cases are encouraging, further evaluation of the proposed procedure is required.

## References

[1] Denis M., Atlan A., Vennat E., Tirlet G., Attal J. P. (2013). White defects on enamel: diagnosis and anatomopathology: two essential factors for proper treatment (part 1). *International Orthodontics*.

[2] Attal J. P., Atlan A., Denis M., Vennat E., Tirlet G. (2014). White spots on enamel: treatment protocol by superficial or deep infiltration (part 2). *International Orthodontics*.

[3] Leppaniemi A., Lukinmaa P. L., Alaluusua S. (2001). Nonfluoride hypomineralizations in the permanent first molars and their impact on the treatment need. *Caries Research*.

[4] Chawla N., Messer L. B., Silva M. (2008). Clinical studies on molar-incisor- hypomineralisation part 1: distribution and putative associations. *European Archives of Paediatric Dentistry*.

[5] Borges A. B., Caneppele T. M. F., Masterson D., Maia L. C. (2017). Is resin infiltration an effective esthetic treatment for enamel development defects and white spot lesions? A systematic review. *Journal of Dentistry*.

[6] Watts A., Addy M. (2001). Tooth discolouration and staining: tooth discolouration and staining: a review of the literature. *British Dental Journal*.

[7] Paris S., Meyer-Lueckel H. (2009). Masking of labial enamel white spot lesions by resin infiltration—a clinical report. *Quintessence International*.

[8] Carvalho L. D., Bernardon J. K., Bruzi G., Andrada M. A. C., Vieira L. C. C. (2013). Hypoplastic enamel treatment in permanent anterior teeth of a child. *Operative Dentistry*.

[9] Muñoz M. A., Arana-Gordillo L. A., Gomes G. M. (2013). Alternative esthetic management of fluorosis and hypoplasia stains: blending effect obtained with resin infiltration techniques. *Journal of Esthetic and Restorative Dentistry*.

[10] Kielbassa A. M., Ulrich I., Treven L., Mueller J. (2010). An updated review on the resin infiltration technique of incipient proximal enamel lesions. *Medicine in Evolution*.

[11] Paris S., Schwendicke F., Keltsch J., Dörfer C., Meyer-Lueckel H. (2013). Masking of white spot lesions by resin infiltration in vitro. *Journal of Dentistry*.

[12] Andreasen J. O., Sundström B., Ravn J. J. (1971). The effect of traumatic injuries to primary teeth on their permanent successors. *European Journal of Oral Sciences*.

[13] Thylstrup A., Andreasen J. O. (1977). The influence of traumatic intrusion of primary teeth on their permanent successors in monkeys a macroscopic, polarized light and scanning electron microscopic study. *Journal of Oral Pathology & Medicine*.

[14] Jälevik B., Norén J. G. (2000). Enamel hypomineralization of permanent first molars: a morphological study and survey of possible aetiological factors. *International Journal of Paediatric Dentistry*.

[15] Fagrell T. G., Dietz W., Jälevik B., Norén J. G. (2010). Chemical, mechanical and morphological properties of hypomineralized enamel of permanent first molars. *Acta Odontologica Scandinavica*.

[16] Fagrell T. G., Salmon P., Melin L., Norén J. G. (2013). Onset of molar incisor hypomineralization (MIH). *Swedish Dental Journal*.

[17] Schneider L. F. J., Cavalcante L. M., Consani S., Ferracane J. L. (2009). Effect of co-initiator ratio on the polymer properties of experimental resin composites formulated with camphorquinone and phenyl-propanedione. *Dental Materials*.

[18] Maciel D. D. S. A., Caires-Filho A. B., Fernandez-Garcia M., Anauate-Netto C., Alonso R. C. B. (2018). Effect of camphorquinone concentration in physical-mechanical properties of experimental flowable resin composites. *BioMed Research International*.

[19] De Geus J. L., Wambier L. M., Kossatz S., Loguercio A. D., Reis A. (2016). At-home vs in-office bleaching: a systematic review and meta-analysis. *Operative Dentistry*.

[20] Hamama H. H. (2013). FOCAL bleaching technique. *Journal of Cosmetic Dentistry*.

[21] Horuztepe S. A., Baseren M. (2017). Effect of resin infiltration on the color and microhardness of bleached white-spot lesions in bovine enamel (an in vitro study). *Journal of Esthetic and Restorative Dentistry*.

